# The Role of Nutrition and Forest-Bathing in the Physical Rehabilitation of Physically Inactive Patients: From the Molecular Aspects to New Nature-Inspired Techniques

**DOI:** 10.3390/ijerph20010793

**Published:** 2022-12-31

**Authors:** Steven Baker, Melinda Gilhen-Baker, Giovanni N. Roviello

**Affiliations:** 1 Compete Physiotherapy and Rehabilitation Centre, Unit 1 Bridge Mill—Cowan Bridge, Carnforth LA6 2HS, UK; 2 National Trust, Bowe Barn, Borrowdale Road, Keswick CA12 5UP, UK; 3 Institute of Biostructures and Bioimaging, Italian National Council for Research (IBB-CNR), Area di Ricerca Site and Headquarters, Via Pietro Castellino 111, 80131 Naples, Italy

**Keywords:** physiotherapeutic management, nature-inspired, biotechnology, diet, biomolecular mechanisms

## Abstract

Physical rehabilitation plays a fundamental role in the management of individuals with disabilities associated with age-related muscle loss or affected by catastrophic conditions such as trauma, surgery, cancer or other severe pathologies. These events have in common an extended period of physical inactivity. Patients who undergo prolonged bed rest often present with a number of complications; for example, muscle loss that can exacerbate existing conditions determined by sarcopenia, which in turn greatly limits physical functions. The main scope of this work is to summarize certain key strategies for the physiotherapeutic management of physically inactive patients, regardless of the reason behind their prolonged bed rest, with a particular focus on physical rehabilitation, nutrition and forest-bathing. The importance of correct nutrition in counter-acting the loss of muscle mass and consequent function is explored alongside a description of the main nutrients that are needed for muscle regeneration. From a biomolecular perspective, some specific molecular mechanisms associated with physical rehabilitation are also reported not only in the context of physical therapy, but also within nature-inspired techniques, such as forest-bathing as well as body self-healing. Combining a targeted physiotherapeutic approach with an appropriate diet as well as nature-based therapy could thus help with the recovery of bed ridden patients.

## 1. Introduction

Patients are typically classified as physically inactive [1,2,3,4,5,6,7,8] if they spend less than two and a half hours per week participating in activities of moderate intensity. Given this definition, the population within Western countries is becoming more and more at risk of falling into this category due to an increase in sedentary behavior. Sedentary behavior is typically linked to poor lifestyle habits, and in particular, a lack of exercise that in turn is associated with a number of negative impacts on metabolic and cardio-vascular functions correlated with an increased risk of developing various disabilities in older adults [9].

Several factors may also determine involuntary physical inactivity. Such causes include cancer, trauma, and prolonged hospitalization after surgery [10,11,12,13]. Among the severe illnesses leading to physical inactivity, cancer [14,15,16] has been considered a highly socially relevant pathology affecting the general population and accounting for one in six deaths over the globe (as reported at https://www.cancer.org/research/cancer-facts-statistics/all-cancer-facts-figures/cancer-facts-figures-2022.html Cancer Facts & Figures 2022, accessed on 22 September 2022). Sadly, cancer can also greatly impact the survivor’s ability to engage in physical activity. This was observed particularly in adult survivors of childhood acute lymphoblastic leukemia [17], who have been found to be less physically active than the wider population [13]. Viral infections can also lead to physical inactivity as observed in the case of COVID-19 survivors [18,19,20,21,22]. These were found to remain physically inactive after recovery [23,24]. Several pathological conditions may significantly limit physical function and in turn, physical inactivity contributes to an increased risk of cardiovascular disease in patients. For example, approximately half of rheumatoid arthritis patients were found physically inactive despite physical activity being strongly encouraged in patients affected by this chronic autoimmune disease to comply with recommendations to prevent cardiovascular morbidity and to improve patients’ quality of life [25].

Following a prolonged period of bed rest, numerous and often interrelated complications may typically manifest. One of the first to occur is muscle loss [26,27], a serious condition that can exacerbate existing problems determined by sarcopenia [28,29,30] which in turn may cause disabilities, especially in older adults [31]. In fact, older patients lose lean tissue and strength very rapidly during prolonged periods of lying or sitting when compared to the young [31]. In this context, physiotherapeutic rehabilitation [32,33,34,35] is a fundamental first-line strategy for the management of patients with a history of physical inactivity for whom one of the consequences has been physical function loss. An exercise program which includes both strength training and aerobic exercise is recommended to rebuild muscle strength and recover overall function. Focus should be placed on activities of daily living (ADL) to help bring the patient back into a healthy routine. This, combined with tailored goal setting facilitated by the physiotherapist, will aid with the behavioral changes necessary to incorporate weekly exercise and ultimate recovery. Physiotherapy may thus have beneficial effects in the activation of the body’s self-healing capabilities and the recovery of muscle function. However, to improve the patient’s chance of recovery, it should also be accompanied by a specific nutrition scheme that includes high-quality proteins with the main purpose of limiting muscle loss, as well as nature-inspired strategies which play a pivotal role in shortening healing times, improving mental health and stimulating the immune system [31]. All this being premised, herein we aim at reviewing some of the main strategies that can be employed in the management of physically inactive individuals regardless of the factors causing their prolonged bed rest. Another aim of this work consists in the discussion of some molecular mechanisms associated with physical rehabilitation, as well as the role of physiotherapy, associated with nature-based therapies, such as ‘forest bathing’ [36,37,38,39,40] and an appropriate nutrition in boosting the intrinsic body’s ability to self-heal.

## 2. Methods

For the preparation of the current review, we explored the recent scientific literature (articles in English published in the period 1 January 1990–20 October 2022) relating to themes reported above in this section, as well as the keywords ‘physiotherapeutic management’; ‘nature-inspired’; ‘biotechnology’; ‘diet’; ‘biomolecular mechanisms’. In these searches, we made use of scientific search engines, such as PubMed and Google Scholar excluding retracted articles and non-English publications. Out of the more than 200 articles examined, we selected the 102 discussed here below, after exclusion of those not written in English, published before January 1992 or after 20 October 2022, whose full text was not found, or with research directions not related to the scope of our work.

## 3. Results and Discussion

**Figure 1:**
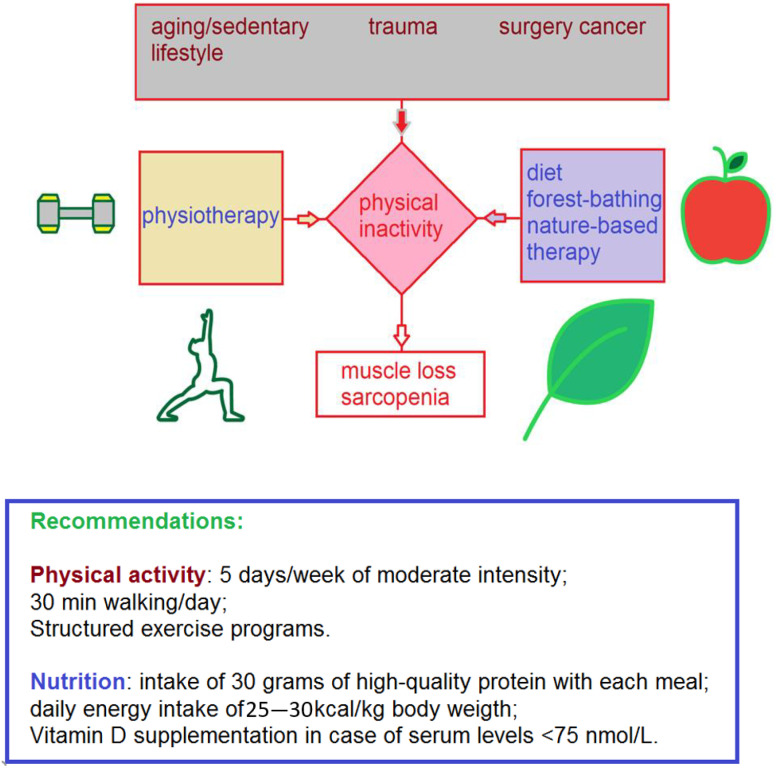
Schematic description of the main issue, including the complications of multicausal physical inactivity, as well as the patients’ main management strategies and recommendations illustrated in this work.

The progression of aging in patients who have suffered catastrophic events including injury, cancer, or other severe illnesses, as well as individuals who simply have a more sedentary lifestyle may result in prolonged physical inactivity (Figure 1) which is linked to protein catabolism and consequent lean body mass loss; a typical condition associated with sarcopenia [41].

For example, a study conducted on older adults showed that 5 days of bed rest are enough to significantly decrease leg lean tissue mass and knee extensor strength [42]. This is determined by a chronic imbalance between the muscle protein catabolism and their new synthesis which is found during a prolonged period of physical inactivity accompanied by several pathological or traumatic conditions which may also highly accelerate the rate of muscle protein breakdown [41]. Interestingly, there is a catabolic interaction between hypercortisolemia and physical inactivity as demonstrated in studies conducted on subjects challenged with 12 h of hypercortisolemia before and after two weeks of bed rest [41]. In particular, cortisol was infused over this period at concentrations similar to those observed in blood after severe trauma (i.e., ~900 nmol/L). Before inactivity, the cortisol challenge did not enhance muscle catabolism with respect to fasting alone. However, after two weeks of inactivity, the same hypercortisolemic challenge not only led to higher levels of protein breakdown but also negatively impacted net muscle protein synthesis [41].

The physiotherapist plays an important role in managing patients with sarcopenia (Figure 1) as they may benefit greatly, regardless of age, from a mixed physiotherapy program which can result in improved gait speed and improved functioning for day to day activities, even with a treatment period as brief as two weeks [42]. The components of such a program vary depending on the patient’s particular situation but focusing on resistance exercise training, aerobic exercise training, mobility training, and functional activities of daily living when combined with shared goal setting has been shown to improve outcomes in patients with sarcopenia [43].

Resistance exercise training is a training method that uses repeated exercise against an external resistance in tandem with appropriate nutrition and recovery strategies to cause cellular adaptations in the muscle tissue which may result in hypertrophy and strength of the exercised muscle [44]. Muscular strength can be defined as the ability to exert a force on an external resistance [45]. Training methods to increase strength via resistance exercise training in humans have been demonstrated to increase treatment outcomes and decrease injury risk [46,47]. The American College of Sports Medicine (ACSM) recommends a 12-week period of resistance training consisting of 3 sessions per week to facilitate these adaptations [46]. Additionally, strength training literature suggests that a training dose of 3–4 sets of 6–12 repetitions is sufficient to develop hypertrophy of the exercised muscle whilst a lower number of repetitions against a heavier resistance is more likely to facilitate a quicker adaptation in strength outcomes [44]. This training theory must be balanced with the needs of the recovering patient who may not initially be able to undertake these prescriptions due to illness or injury [45]. Despite the ACSM recommendations of a 12 week period to allow suitable adaptations, some changes were observed in as a little as 2 weeks and so, even if only a short period of exercise is possible due to issues such as access to a suitably trained provider, it may be beneficial to begin the training with reduced volume [47]. Strength training programs should focus on improving the ability of the major muscle groups associated with locomotion and daily functional tasks, such as the ability to get out of a chair and squat down, to improve function in the post bed-ridden patient [48]. Ideally, a resistance training program for older individuals should include 2–3 sets of 1–2 multi-joint exercises per major muscle group, achieving intensities of ~80% of 1 repetition maximum (1RM), 2–3 times per week, including power exercises performed at higher velocities in concentric movements with moderate intensities (for example ~50% of 1RM) [48].

Programs focused on these areas have also been shown to reduce the mortality risk associated with serious injury due to falls and subsequent hospitalization [49].

Interestingly, the previously-mentioned ACSM defines aerobic exercise training as any activity that uses large muscle groups which can be maintained continuously and is rhythmic in nature [50]. Improved aerobic function has been linked to a decreased risk of chronic disease and mortality in the literature and is recommended by most governments in the developed world [50,51]. This form of exercise uses low load repetitive movements, such as walking, jogging, cycling and aerobics to create an exercise load on the body that can be met with the cardiovascular system. The World Health Organization (WHO) recommends 150 min per week of moderate activity or 75 min of vigorous activity per week to decrease the risk of chronic diseases [51]. The recovering patients who have undertaken a period of hospitalization due to illness, such as cardiac disease, should start with modest periods of aerobic training. For example, walking in bouts of 3–5 min and progressing as tolerated to a duration of 10–15 min daily is recommended until such time as their physical capacity has improved enough to be able to undertake the recommendations for a healthy adult [50]. The ACSM, WHO and the Chief Medical Officer of the United Kingdom also recommend combining both resistance exercise training and aerobic activity weekly for all adults [50,51,52]. Of late, there is a paucity of research investigating the effects of a combined strength and aerobic program on individuals with sarcopenia. Research by Bower, Schuler & Adams, 2015 has shown that programs consisting of resistance exercise training and aerobic training are more effective in decreasing the effects of muscle loss due to sarcopenia than resistance training alone [53]. It also demonstrated that the older adult is just as likely to improve muscle function as a 20 year old up until the seventh decade in life [53]. However, once past 80 years of age, aerobic training may be more effective in inducing muscle regeneration as it is hypothesized that it is more effective at increasing capillarization of the working muscles than resistance training [53]. From a biomolecular point of view, animal studies indicated PPARgamma coactivator 1α (PGC-1α), an important exercise-induced transcription factor regulating mitochondrial biogenesis, may be responsible for many of the intracellular improvements associated with exercise training in sarcopenia [53].

Whilst it is important to focus on exercise methods to enhance skeletal muscle function and regeneration, physiotherapy programs should also encompass elements that examine individual performance of patient specific functional tasks [54]. Activities of daily living is an area of patient specific activity that adults use to manage their daily life [55]. These are tasks that a patient may not be able to perform due to muscle loss and loss of skill due to forced bed rest and resultant sarcopenia [54]. This requires the physiotherapist to create functional exercise activities and tasks to train the patient to regain these functional, individual goals [56]. These tasks, due to their personal nature, are crucial to the individual’s ability to regain their independence and should have primacy in any mixed therapy program so as to expediently return the patient to pre-bed rest levels and to prevent secondary complications of a physical and mental health nature [57]. From a physical perspective, exercise may reduce falls by 21%, especially if it includes challenging balance training for more than 3 h/week [57].

Functional and community mobility can be classified as instrumental activities of daily life (IADL) and was a concept introduced by Katz et al. in the 1950’s [58]. This is a crucial area of development for all post bed-ridden individuals regardless of their mode of locomotion to ensure the patients regain and maintain their functional independence [59]. Various assistive devices can be used to assist mobility from a cane to wheeled walkers and wheelchairs. IADL training should focus first and foremost on the safety of the patient while moving and then to create improvements where they can be achieved via strength training and walking training to improve independence and decrease the risk of falling [60]. Additionally, measures of strength may be a useful indicator in the prediction of those at increased risk of falling and in need of a fall prevention program [61].

The Cambridge online dictionary defines goal setting as ‘the process of deciding what you want to achieve or what you want someone else to achieve over a particular period’ (https://dictionary.cambridge.org/dictionary/english/goal-setting accessed on 19 October 2022). In physiotherapy it can be used to unite the physiotherapist and patient in the creation of a treatment plan that may improve outcomes in strength, range of motion and balance in the recovering patient [62]. Goal setting with the sarcopenic patient has been described by some authors as imperative with regards to resistance training and may be most beneficial when provided by healthcare professionals, such as physiotherapists [63]. However, despite its widespread adoption, goal setting in physiotherapy is reported by some authors as a complex process due to a lack of patient knowledge on meaningful treatment goals and so care must be taken in the consultation process to ensure patients have the necessary information [64]. Goal setting is also one of a range of activities that has been reported in the literature to be effective for facilitating behavioral change in sedentary individuals and as a result, may improve the economic burden of chronic disease [65].

Physical inactivity, together with excessive weight gain and increased body fat mass, heightens the risk of several other health problems, such as hypertension [66]. Participating in physical activity of moderate intensity on a regular basis was also shown to reduce body fat mass and promote better functioning of the cardiovascular, renal, and nervous systems, with the added benefit of regulating blood pressure [66]. In fact, physical inactivity affects 20% of the adult population over the globe and increases the risk of hypertension by 30%. Physical activity however, reverses hypertension associated with body fat mass, and has a comparable effect to pharmacologic monotherapy. For the obese patient with hypertension, the exercise program is modified and progressed based on the physiotherapist’s initial assessment of the individual’s capacity and the physiotherapist’s and patient’s goals [66]. Physiotherapists have the clinical competency to influence physical activity behaviors in their patients as they act not only as rehabilitators but also as promoters. They possess the skills and knowledge necessary to facilitate this behavioral change as well as to promote adherence to physiotherapy itself [67]. In promoting physical activity as part of their practice, physiotherapists use a number of techniques to help modify behavior. In a recent study, ‘social support’ [68] was identified as the behavioral change technique most frequently used by physiotherapists [69]. By adding to that a more conducive environment to healing, such as a relaxing natural green space, the successful rehabilitation of the patient is even more likely.

As for the biological mechanisms involved, adult stem cells [70,71,72,73,74] determine the human body’s ability to continuously regenerate after traumatic events and injuries. These are undifferentiated cells endowed with the ability to self-renew indefinitely or differentiate in order to maintain the integrity of the tissues. Interacting with the extracellular matrix, adult stem cells are able to influence some cytoskeleton mechanical properties, such as remodeling and plasticity, that are crucial in wound healing. Moreover, physical as well as mechanical stimuli are known to modulate the fate of stem cells in their own niche and are able to boost the intrinsic ability the body has to self-heal, without requiring stem cell transplantation or any other more invasive treatments [75]. From a biomolecular perspective, the secretion of growth factors from bone marrow mesenchymal stem cells (BM-MSCs) [76,77,78,79] following artificial stimuli may regulate the inflammatory environment by promoting the expression of M2 macrophages and inhibiting the expression of M1 macrophages [80], which results in an enhanced wound healing effect [81].

Physical activity leads to benefits for both mental and musculoskeletal health and significantly reduces the risk of type 2 diabetes, cardiovascular diseases and cancer thanks to biological mechanisms that include links between obesity and body mass, changes in metabolism, insulin resistance, and other immune, inflammatory, and hormonal factors [82]. The recommendation of physical activity intervention to preserve mobility in older individuals with sarcopenia and physical frailty includes 5 days a week of moderate intensity physical activity, walking half an hour per day and different structured exercise programs (Figure 1) [59]. Another first line strategy useful to accelerate the healing process after traumatic events and slow sarcopenia is adopting an appropriate diet. In fact, correct nutrition was able to prevent muscle catabolism and promote the anabolic processes as demonstrated in bed rest studies conducted on healthy volunteers [41]. These scientific investigations highlighted the role of dietary-derived amino acids as prerequisites necessary to induce the new synthesis of muscle proteins and consequent muscle building in the volunteers examined. In other words, the amino acid supplementation accompanied by an intake of approximately 30 g of high-quality proteins with each meal [31] are fundamental in the maintenance of muscle mass and related strength during age-related and medically mandated physical inactivity [41]. Moreover, a daily total energy intake of 25–30 kcal/kg body weight is recommended. Vitamin D levels should also be monitored regularly, and supplementation should be considered if serum concentrations of 25-hydroxyvitamin D are found lower than 75 nmol/L [59].

**Figure 2:**
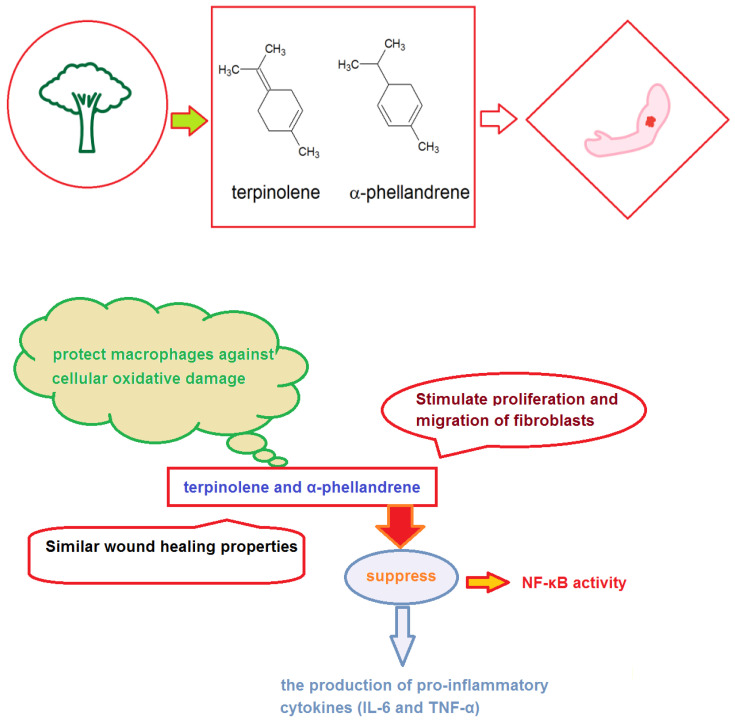
Some plant-produced volatile organic compounds involved in wound healing and their biomolecular mechanism.

As already alluded to, ensuring that a patient’s mental health is looked after and that they are exercising in an environment that is conducive to their healing will go a long way in helping them to achieve their health goals. The importance of spending time in nature to promote healing after traumatic events is increasingly well documented. Forests in particular provide the ideal location for physical exercise and patients should be encouraged to walk in a beautiful and relaxing natural setting, rather than staying indoors or driving through a stressful urban environment. Forest bathing, or forest therapy, has a key role in facilitating healing post-trauma which is one of the reasons why this nature-inspired therapy [83,84] is gaining an ever-growing scientific interest for its many important therapeutic implications on both our mental and physical health [85]. Indeed, although it has in the past been common for people to seek out the forest for medicinal purposes, in 1982 the government of Japan began recommending the practice of Shinrin-Yoku to its citizens who still take time weekly to walk in the local forests. The benefits gained mainly stem from the many plant-emitted volatile molecules that once inhaled cause invigorating effects on the human immune system [85,86,87]. Interestingly, some plant-derived volatile organic compounds (VOCs) [88,89,90,91,92], such as terpinolene and α-phellandrene (Figure 2), were also shown in vitro to promote the wound healing process through their ability to attenuate oxidative stress and inflammation [93].

These compounds, despite their identical molecular weight, show some different physical properties with terpinolene being more soluble in water but less volatile (Table 1).

As for their biological properties, terpinolene was endowed with a higher antioxidant activity but the stimulatory effect on migration and proliferation of L929 fibroblasts was comparable for both isomeric compounds (Table 2). In particular, they were found able to protect macrophages against cellular oxidative damage, stimulated proliferation and migration of fibroblasts and suppressed both the activity of Nuclear factor-κB (NF-kB), a ubiquitous transcription factor involved in different inflammatory and immune responses, as well as the production of pro-inflammatory cytokines, such as TNF-α and IL-6 (Figure 2) [93].

Sessions of forest bathing can be tailored to the specific causes determining the physical inactivity, whether it be cancer or states of depression, by seeking out particular trees for the specific composition of their medicinal VOCs [94]. It is also worth bearing in mind that spending time immersed in nature, inhaling biogenic VOCs into the lungs or absorbing them through the sebaceous glands present on the skin, leads to decreased cortisol levels and promotes the body’s ability to heal regardless of the underlying issues [95].

There is clear evidence on the link between nature and human health and although our relationship to the forests seems to be more tenuous in this technological age, they have long been our main source of medicines. Most of our modern pharmaceuticals still make use of or mimic medicinal and therapeutic compounds which are extracted from fungi, plants or marine compounds [96,97,98,99,100]. It is perhaps our long connection with nature which makes forests ideal locations to de-stress but these benefits are not limited to woodlands. It has been found that humans also benefit enormously from the positive effects of spending time near clean rivers [101,102]. Good quality river water together with thriving biodiversity and a healthy riparian habitat structure combine to create a beneficial space for people to enjoy exercise or sessions of relaxation. Similar to the documented effect of being immersed in or even being subjected to images of nature on the mental state of patients, there is also quantitative evidence for the relaxing effect of the sounds made by rivers. As with trees in a forest setting, riparian vegetation can also boost the human immune system with its plant-emitted volatile organic compounds. River water also carries plant metabolites that are known ingredients for traditional medicines which induce, among other beneficial effects, antipsoriatic healing [101,102]. Including nature-based therapies in any recovery program is a simple and effective way to help patients by accelerating their physical and mental wellbeing which in turn will encourage them to follow the protocols set by their health-care provider. Protecting river ecosystems and improving water quality are therefore challenges worth facing to help preserve their ability to improve psychological and physical wellness [101,102]. Overall, safeguarding and improving the state of our natural spaces, such as rivers and woodlands, not only provides economic, social, and environmental advantages but also highly encourages increased physical activity while bringing their own therapeutic benefits to the wider population [82].

## 4. Conclusions

In conclusion, regardless of the etiology leading to their prolonged physical inactivity, physiotherapy exerts a key role in the management of physically inactive patients with functional losses caused by a plethora of circumstances, including catastrophic events, such as trauma, surgery, and cancer, as well as aging and having a sedentary lifestyle. This lack of physical activity, which is so beneficial to our health and wellbeing, is clearly the cause of many associated mental, emotional, social and physiological ailments. Prolonged hospitalization or bed rest after traumatic events may have debilitating effects that are correlated with sarcopenia, or muscle loss, a condition that in turn may cause the loss of important physical functions or in the most severe cases physical disability. It is advisable that nature-inspired therapists and nutrition specialists work together with physiotherapists for the holistic management of physically inactive patients regardless of the underlying issues. This should include a tailored exercise program which combines resistance training and aerobic exercise which focuses on, at least at the beginning, re-establishing activities of daily living. Moreover, 5 days per week of moderate intensity physical activity, as well as walking half an hour per day and taking part in different structured exercise programs are among the recommendations for physical activity intervention to preserve mobility in older individuals with sarcopenia and physical frailty. From a more biological perspective, adult stem cells and different proteins, such as growth factors, are involved in biomolecular mechanisms linked to regeneration which are in need of further scientific investigation. Physiotherapeutic exercises of moderate intensity accompanied by a specific nutrition plan rich in high-quality proteins and other nutrients, and nature-based approaches, such as forest therapy with patient exposure to biogenic volatile organic compounds, are all strategies that concur to further aid in the physical rehabilitation process of patients with sarcopenia. The added mental health benefits of being supported by an attentive physiotherapist while in a relaxing and inspiring natural setting can only help insure a successful recovery. Environmental improvements in forest and riparian ecosystems should then not simply be regarded as investments in our landscape and its biodiversity but also in our physical health and wellbeing. Woodlands and rivers provide ideal locations in which to perform physical activity as well as environments that can increase our overall health and life expectancy. As per Dr. Diana Beresford Kroeger’s Bioplan concept, it would thus be well worth looking into more ways of integrating nature into therapeutic spaces to facilitate such combined healing practices and ensuring the future of our health as well as that of the planet [94].

## Figures and Tables

**Table 1:** Some chemical and physical properties of terpinolene and α-phellandrene ^1^.

Compound	Molecular Weight [g/mol]	Solubility in Water [mg/L]	Vapor Pressure [mmHg]
terpinolene	136.23	9.5	0.74
α-phellandrene	136.23	insoluble	1.4

^1^ Data retrieved from PubChem (https://pubchem.ncbi.nlm.nih.gov/ accessed on 5 December 2022).

**Table 2:** Comparison of the antioxidant activities and maximum stimulatory effects on L929 fibroblast migration and proliferation of terpinolene and α-phellandrene [94].

Compound	Antioxidant Activity (IC_50_ μM) ^1^ ± SD ^2^	Stimulatory Effect at 200 μM on L929 Fibroblasts (%) ± SD ^2^
terpinolene	409.4 ± 1.6	36.3 ± 4.8
α-phellandrene	216.9 ± 5.7	39.1 ± 3.9

^1^ as evaluated for NO scavenging activity [94]. ^2^ SD stands for Standard Deviation.

## Data Availability

Not applicable.
